# Expatriation stressors and the well-being of accompanying partners: a commonality analysis approach

**DOI:** 10.3389/fpsyg.2025.1607178

**Published:** 2025-06-04

**Authors:** Katja Herrmann Aegerter, Andrea H. Meyer, Jens Gaab, Yoon Phaik Ooi

**Affiliations:** ^1^Division of Clinical Psychology and Psychotherapy, Faculty of Psychology, University of Basel, Basel, Switzerland; ^2^Department of Developmental Psychiatry, Institute of Mental Health, Singapore, Singapore

**Keywords:** expatriation stressors, accompanying partners, international assignments, commonality analysis, well-being

## Abstract

**Introduction:**

This cross-sectional study aims to explore the unique and shared effects of non-work expatriation stressors on the well-being of expatriate partners and spouses who relocate on a regular basis.

**Methods:**

A cohort of 207 internationally mobile adults was recruited through international associations, foreign ministries, social media, and personal networks. Participants completed a quantitative online questionnaire that assessed various psychological factors. We employed commonality analysis to evaluate the unique and joint impact of perceived stress, perceived social support, isolation, and perceived cultural distance on partner well-being, using validated psychological scales.

**Results:**

Perceived stress proved to be the most impactful unique contributor to partner well-being, while isolation emerged as the second strongest unique predictor. Perceived social support showed the most substantial combined effect with stress and isolation. The variance explained by perceived cultural distance was marginal, suggesting that stress and isolation are more influential factors in this population. The control variables (age, gender, duration of residence in the host country, and frequency of relocation) showed no significant contribution in combination with the stressors.

**Discussion:**

Building on the findings of existing research, these results provide further support for the need for tailored interventions to promote the well-being of expatriate partners. Practical implications include involving partners in pre-assignment screening processes, investing in structured social support systems to reduce isolation, and developing comprehensive, culturally sensitive policies that address the range of challenges faced by expatriate partners.

## Introduction

As markets have become more globalized over the past decades, modes of employment have similarly expanded across borders. Consequently, the expatriation of employees has become increasingly common, with an estimated 87.5 million employees transferring abroad for work reasons (Albien and Ruedin, 2023). In this paper, we used the term expatriation to refer to the international deployment of employees, who—in contrast to other populations on the move (e.g., long-term immigrants, refugees, asylum seekers, exchange students)—voluntarily relocate abroad for a preset amount of time (Sterle et al., 2018a) to sustain their careers, i.e., in the private and public sectors, nongovernmental or governmental organizations, or academia (Berry et al., 2011), often accompanied by their partner and/or children (Caligiuri and Bonache, 2016). McNulty (2014) defines an expatriate family as “married, de facto, live-in, or long-term partners of the opposite or same sex, with or without children, with family members that reside in one or many locations; and legally separated or divorced (single) adults with children, with family members that resided in one or many locations” (p. 339).

Expatriation comes with both assets and drawbacks, which permeate multiple life areas, not only for expatriate employees but also for their accompanying spouses and partners. Generally, moving is perceived as stressful, resulting in a heightened baseline of stress perceptions for expatriate families (Cheung and Wong, 2022). Numerous studies have demonstrated that the levels of stress experienced by expatriates are increased (Anderzén and Arnetz, 1997; Aswegen, 2009; Berry, 2006; Brown, 2008; Doki et al., 2018; Moyle and Parkes, 1999; Riemer, 2000; Silbiger and Pines, 2014), which can adversely affect their well-being (Anderzén and Arnetz, 1997), and create spillover effects on other family members or crossover into other life areas (Sterle et al., 2018a).

Despite receiving less scholarly attention, the experience of expatriation for accompanying spouses and partners can be even more complex and multifaceted. Unlike expatriate employees, who typically enter pre-structured work environments, accompanying partners must rebuild their daily lives independently in unfamiliar settings, managing essential logistics such as housing, transportation, healthcare access, and schooling—often in a foreign language and cultural context (Andreason, 2008). This responsibility, coupled with limited initial support, can lead to significant social isolation and chronic stress (Brown, 2008; Kupka and Cathro, 2007). While expatriate employees engage daily with colleagues and benefit from organizational support, accompanying partners frequently experience loneliness, far from extended family or long-standing friendships, and with limited opportunities for meaningful social interaction (Sterle et al., 2018a). Many partners are also forced to give up their professional careers, which can severely impact their identity, sense of purpose, and self-worth (Rosenbusch and Cseh, 2012). The transition from an independent professional to a dependent homemaker or informal caregiver represents a dramatic change in self-perception and daily routine. These challenges are compounded by uncertainty about the future—employment prospects, potential career gaps, and the implications of further relocations—creating a persistent sense of instability (Rosenbusch et al., 2015; Sterle et al., 2018a).

At the same time, accompanying partners play a key role in supporting expatriate employees and thus contributing, also through crossover effects, to successful expatriation (Biswas et al., 2022; Shaffer and Wan, 2020). The inability of spouses to adjust is frequently cited as the primary reason for early abortions of an assignment (Andreason, 2008), with a premature termination leading to considerable financial costs for the employer (Aracı, 2015; Doki et al., 2018) and even greater pressure for the expatriate employee and their accompanying family. Given the systemic influence of stress on the well-being of expatriate spouses and partners, and its well-documented crossover effects on the employee and children (Sterle et al., 2018a), as well as the threat it poses to the overall success of an expatriation (Haslberger and Brewster, 2009), it is crucial to broaden our understanding of the specific stressors affecting expatriate spouses and partners.

Our study aims to identify and empirically examine key stressors that significantly contribute to psychological strain in expatriate spouses and partners, with the goal of extending and improving existing support measures. Rather than using a formalized theoretical framework, we drew our insights from transactional stress theory (Lazarus and Folkman, 1984; Wurtz, 2022), and acculturation theory (Berry, 2006), all of which emphasize the interplay of individual perceptions and environmental stressors in shaping psychological well-being. Within this conceptual foundation and guided by the current literature on stressors in expatriate partners and spouses (Flachenecker and Gröschke, 2021; Rosenbusch et al., 2015; Sterle et al., 2018a), we chose to focus on four stressors—perceived stress, perceived social support, isolation, and perceived cultural distance. Those were selected based on both theoretical relevance and empirical evidence in the expatriation literature.

While we recognize that individual traits such as stress resilience are acknowledged mediators for mental health (Jones et al., 2023) or adjustment (Reed et al., 2023) of expatriate family members, this study focuses on situational stressors that are potentially modifiable through support interventions. These stressors often manifest through a range of physical, emotional, cognitive, or behavioral symptoms. Research indicates a raised experience of general stress among expatriates (Silbiger and Pines, 2014), which can trigger psychophysiological responses, including elevated levels of certain stress hormones, increased cardiovascular risk, and decreased mental well-being, as shown in a comparative study of expatriates and a control group (Anderzén and Arnetz, 1997). Emotional symptoms frequently manifest as anxiety, irritability, or pervasive feelings of sadness (Cangià, 2017; Kanstrén and Mäkelä, 2020; Perone et al., 2008) in expatriate partners and spouses. Cognitive symptoms may be evidenced by role or identity confusion or value-related dilemmas (Osland, 2000; Rosenbusch and Cseh, 2012). From a behavioral perspective, individuals may exhibit withdrawal, alterations in sleep or eating patterns, and an increased reliance on alcohol consumption, particularly among male expatriates (Burkholder et al., 2010; Rosenbusch et al., 2015; Takeuchi, 2010; Wurtz, 2018). The stress experienced can be acute, arising from isolated incidents related to daily living challenges, or chronic due to more enduring factors, such as a lack of close friends to confide in or the inability to spend quality time with one’s partner due to increased work commitments of the expatriate employee (Brown, 2008). While the original culture shock model suggests that stress perceptions typically happen in several stages and change over time in a host country (Oberg, 1960), the linearity of stress perception has not been substantiated (Brown, 2008).

Isolation and the lack of perceived social support are interconnected. With a transition to a foreign country, the partners and spouses of the expatriate employee may feel stressed due to feelings of isolation (Brown, 2008). Isolation can result from a lack of time spent with one’s partner and a lack of close friends in the host country (Sterle et al., 2018a). In particular, immediately after moving to a new country, many expatriate workers and partners experience loneliness and isolation (Bahn, 2015). However, even several months after the move, expatriate partners continue to worry about losing contact with friends and family in their home countries due to the relocation (Forster, 1997). Social support can be crucial for the adjustment of expatriate spouses (Copeland and Norell, 2002). Notably, expatriates’ support from companies and families was found to be significantly related to their cross-cultural adjustment (Caligiuri et al., 1999). In reality, the expatriate community in a host country is often the only source of support for expatriate families (Kupka and Cathro, 2007). However, this network is not predetermined; it must first be established upon arrival at a new location, and its size and accessibility may vary depending on the type of posting.

Perceived cultural distance refers to the differences in specific domains between the previous host/home country and the new country of assignment. These domains include the environment, daily living, norms, language, and social contacts (Demes and Geeraert, 2014). The stronger the differences are, the lower an expatriate’s sense of agency and familiarity, and the greater their psychological strain. As spouses and partners face these stress factors more frequently due to their increased exposure to everyday challenges, they may experience higher levels of stress. This vulnerability is closely related to the concept of acculturative stress, which Berry (2006) defined as “a response by individuals to life events (that are rooted in intercultural contact)” (Berry et al., 2002, p. 362), and is originally rooted in the idea of culture shock (Oberg, 1960). The reactions to cultural loss and uncertainty about the future can result in depression and anxiety (Berry, 2006). In an immigrant population, acculturation stress can contribute to negative mental (Salas-Wright et al., 2015) and physical health (Gonzalez-Guarda et al., 2021). Galchenko and van de Vijver (2007) found that exchange students who experienced a greater cultural distance between their home and host countries showed lower adjustment. For expatriate families with children, the level of cultural distance is a crucial factor in the decision to accept or reject an expatriate assignment (Dupuis et al., 2008).

Collectively, in this paper, the combined influence of these four stressors (perceived stress, perceived social support, isolation, perceived cultural distance) is referred to as expatriation stress. This provides a parsimonious yet comprehensive way to summarize the primary source of stress for expatriate partners. While previous research has identified various individual stressors such as role loss and identity disruption (Kupka and Cathro, 2007), financial strain and career disruption (Shaffer et al., 2012), our focus narrows to stressors that are widely applicable across partner demographics and can be addressed through organizational or psychosocial support. These stressors have been empirically linked to diminished partner well-being, and, through crossover effects, to the adjustment, well-being, and success of the expatriate employee and accompanying children (Haslberger and Brewster, 2009). Addressing the stressors faced by partners can lead to better preparation for expatriate assignments, potentially reducing the significant costs incurred by sending organizations due to premature terminations of an international assignment (Aracı, 2015). We also recognize that the mentioned additional stressors can intersect and exacerbate the core stressors we examine; for example, role loss may lead to feelings of perceived stress and isolation, while career disruption could intensify feelings of cultural distance or perceived social support.

By aligning our selection of stressors with both theoretical principles and empirical evidence, we aim to expand on the literature and make unique contributions to our understanding of expatriate partners’ stress processes (1) by offering a measure to dissect non-work expatriation stress factors and their contribution to the well-being of the expatriate partner and (2) by quantifying the unique and common effects on the well-being of expatriate spouses/partners. Due to the exploratory nature of this study, we abdicate from presenting a predictive hypothesis and instead use commonality analysis to investigate the relative weight of each stress factor. Analyzing the potential influences of each individual stressor on the well-being of expatriate spouses and partners will contribute to an understanding of the effects of expatriation, inform the development and improvement of targeted support mechanisms, and, consequently, may prevent specific expatriation challenges and premature assignment terminations.

## Materials and methods

### Study procedure and sample characteristics

Data from 207 participants (see Table 1) were collected via a cross-sectional, quantitative English online questionnaire hosted on the web server of the University of Basel. As this investigation was part of a more extensive study on the well-being of expatriate families and children, to be included in the study, participants needed to (1) follow a predicted international relocation rhythm of 2 to 6 years for career reasons, (2) have at least one child between the ages of seven and 17, and (3) be proficient in English. Participants were excluded if they had no children, held the passport of their current country of residence, or were long-term immigrants, refugees, asylum seekers, or foreign exchange students. Participants were recruited through international partner and spouse associations, foreign ministries, social media, and personal contacts. Before completing the survey, participants were asked to confirm the inclusion criteria and consent to participate. Their anonymity was ensured because no personal information or IP addresses were collected. No monetary reimbursement was given for participation. The Ethics Committee of Basel University approved the study (Ethics Approval Number: 028–21-2).

**Table 1:** Demographic variables of the study participants (*N* = 207).

Variables	N	%
Gender		
Female	191	92.3
Male	15	7.2
Other	1	0.5
Age		
25–34	2	1.0
35–44	87	42
45–54	105	50.7
55–64	13	6.3
Home Country Region		
Europe	131	63.3
Middle East & North Africa	2	1
Sub-Saharan Africa	2	1
Asia	11	5.3
Australia & Oceania	7	3.4
North America	48	23.2
Central America & Caribbean	4	1.9
South America	2	1
Number of International Relocations		
1–2 times	57	27.5
3–4 times	60	29
Five or more times	90	43.5
Country of Current Residency		
Europe	105	50.7
Middle East & North Africa	13	6.3
Sub-Saharan Africa	14	6.8
Asia	47	22.7
Australia & Oceania	5	2.4
North America	11	5.3
Central America & Caribbean	5	2.4
South America	7	3.4

### Instruments and measures

#### Perceived stress

Perceived stress was assessed using the short 4-item version of the 10-item Perceived Stress Scale (PSS-Short; Cohen et al., 1983). The PSS scale questions, which primarily measure emotional and cognitive symptoms of stress, such as assessing the participant’s ability to control life issues or handle personal problems, were rated on a Likert-type scale ranging from 0 (never) to 4 (very often). The results were summed to provide the total score, with higher scores indicating higher levels of stress perception. The Cronbach’s alpha for the 4-item version of the PSS in this sample was 0.75.

#### Perceived social support

Perceived social support was assessed using the brief 6-item form of the original 14-item Perceived Social Support Questionnaire (F-SozU-K6; Kliem et al., 2015). Statements such as “*I receive a lot of understanding and security from others*” or “*If I am very depressed, I know who to turn to”* were included in the questionnaire and were rated on a 5-point Likert scale ranging from 1 (strongly disagree) to 5 (strongly agree). The summarized results provided the total score, with lower scores representing lower levels and higher scores representing higher levels of perceived social support. The questionnaire was reliable for this sample, with a Cronbach’s alpha of 0.88 for the 6-item version.

#### Isolation

Isolation was measured using the short 3-item version of the original 20-item Loneliness Scale (R-UCLA; Hughes et al., 2004). Responses were rated on a Likert-type scale ranging from 1 (hardly ever) to 3 (often), and the results were summed to provide the total score. The items included the following three questions: “*How often do you feel that you lack companionship?*,” “*How often do you feel left out?*” and “*How often do you feel isolated from others?*.” Higher scores indicated a greater feeling of isolation. In this sample, the Cronbach’s alpha of the 3-item short version was 0.81.

#### Perceived cultural distance

Perceived cultural distance was assessed using the 12-item Brief Perceived Cultural Distance Scale (BPCDS; Demes and Geeraert, 2014). The items included ratings of differences on topics such as natural and social environments, living conditions, practicalities, food and eating, and family life, with responses rated from 1 (very similar) to 7 (very different). The responses were then summarized to obtain final scores. Lower total scores indicated a lower level of perceived cultural distance, and higher scores indicated a greater level of perceived cultural distance between the previous and current countries of residence. The Cronbach’s alpha for the BPCDS for this sample was 0.93.

#### Well-being

Well-being was measured using the 5-item WHO-5 Well-Being Index (Topp et al., 2015). The questionnaire included statements such as “*I have felt cheerful and in good spirits*” or “*I woke up feeling fresh and rested*” and was rated on a 5-point Likert scale from 0 (at no time) to 5 (all of the time). The ratings were summed to provide final scores; lower scores represented lower levels, and higher scores represented higher levels of well-being. In this sample, the Cronbach’s alpha for the WHO-5 was 0.87.

#### Control variables

Based on the evidence in the current literature and to investigate their potential effects on the outcome, we included age, sex, duration of stay in the host country, and number of international relocations (Cieri et al., 1991; Martin, 1995; Stawski et al., 2008; Yalcin-Siedentopf et al., 2021) as covariates.

### Data analysis

The data analysis was carried out in R version 4.3.2, including the R package yhat (Nimon et al., 2023). The data were analyzed using multiple linear regression to examine whether perceived stress, perceived social support, isolation, and perceived cultural distance predicted well-being after controlling for age, gender, duration of stay in the host country, and number of international relocations. In addition, commonality analysis was chosen as the most suitable extension to multiple regression analysis to decompose the multicollinearity of the four predictor variables (Gustavson et al., 2018).

## Result

### Preliminary analysis

The descriptive statistics of all the study variables are presented in Table 2. The assumptions of linearity, normality, multicollinearity, and homoscedasticity of residuals were met for the linear regression, and no significant outliers were detected.

**Table 2:** Descriptive statistics of predictor variables.

Variables	N	Mean	SD	Skewness	Kurtosis
WHO5	207	14.15	4.9	−0.41	−0.3
PSS	207	5.67	2.97	0.32	−0.53
FSozU-K6	207	22.01	5.32	−0.63	−0.15
RUCLA	207	5.44	1.68	0.3	−0.54
BPCDSD	207	49.79	17.19	−0.14	−0.98

WHO5, World Health Organization Well-Being Index; PSS, Perceived Stress Scale; FSozU-K6, Perceived Social Support Questionnaire (short version); RUCLA, Loneliness Scale (short version); BPCDSD, Brief Perceived Cultural Distance Scale.

The results from the correlation analysis (presented in Table 3) indicated moderate negative correlations between partner well-being and perceived stress (*r* = −0.63, *p* < 0.001) and between partner well-being and sense of isolation (*r* = −0.54, *p* < 0.001). A moderate positive correlation was detected between perceived social support (*r* = 0.44, *p* < 0.001) and partner well-being. A weak negative correlation existed between perceived cultural distance and partner well-being (*r* = −0.22, *p* < 0.01). A moderate negative correlation was found between perceived stress and perceived social support (*r* = −0.40, *p* < 0.001) and between perceived social support and isolation (*r* = −0.55, *p* < 0.001). Perceived stress and isolation were moderately positively correlated (*r* = 0.4, *p* < 0.001).

**Table 3:** Correlations between variables.

Variables	WHO5	PSS	FSoz-U-K6	RUCLA	BPCDSD
WHO5	–				
PSS	−0.63***	–			
FSozU-K6	0.44***	−0.40***	–		
RUCLA	−0.54***	0.4***	−0.55***	–	
BPCDSD	−0.22**	0.08	−0.03	0.17*	–

WHO5, World Health Organization Well-Being Index; PSS, Perceived Stress Scale; FSozU-K6, Perceived Social Support Questionnaire (short version); RUCLA, Loneliness Scale (short version); BPCDSD, Brief Perceived Cultural Distance Scale. *p < 0.05, **p < 0.01, ***p < 0.001.

### Relationships among perceived stress, perceived social support, isolation, perceived cultural distance, and well-being

Multiple regression analysis revealed that higher levels of perceived stress, perceived social support, isolation, and perceived cultural distance predict lower partner well-being (see Table 4). All predictors accounted for a total of 53.28% (R^2^) of the variance in partner well-being, *F*(9, 197) = 24.96, *p* < 0.001. Perceived stress (B = −0.76, *p* < 0.001) and isolation (B = −0.84, *p* < 0.001) explained the most variance in partner well-being. Perceived cultural distance (B = −0.04, *p* < 0.01) explained a minor amount of the variance in partner well-being, while perceived social support had a statistically insignificant relationship with well-being. None of the covariates contributed significantly to the variance in partner well-being. The estimated regression coefficients (B), standard errors (SE), standardized regression coefficients (*β*), and significance levels are reported in Table 4.

**Table 4:** Hierarchical multiple regression with perceived stress, perceived social support, isolation, and perceived cultural distance predicting well-being.

Predictor	B	SE	*β*	*p*
PSS	−0.76	0.09	−0.46	0.001***
FSozU-K6	0.07	0.06	0.08	0.19
RUCLA	−0.84	0.18	−0.29	0.001***
BPCDSD	−0.04	0.01	−0.14	0.006**

PSS, Perceived Stress Scale; FSozU-K6, Perceived Social Support Questionnaire (short version); RUCLA, Loneliness Scale (short version); BPCDSD, Brief Perceived Cultural Distance Scale. *p < 0.05, **p < 0.01, ***p < 0.001.

The results from the commonality analysis are presented in Table 5. Perceived stress accounted for the strongest variance in partner well-being, with a unique effect of U = 0.16, a common effect of C = 0.24, and a total contribution T (= U + C) of 0.40, translating into %R^2^ = 30.04% of the total variance in partner well-being explained by perceived stress. Isolation was the second strongest unique predictor of partner well-being, with a unique effect of U = 0.05, a common effect of C = 0.24, and a total contribution of T = 0.30, translating into %R^2^ = 9.88% of the total variance explained by isolation. Sense of social support (U = 0.0040; C = 0.19) and perceived cultural distance (U = 0.02; C = 0.03) explained only a minor amount of the total variance in partner well-being. In addition, the most substantial contributions for shared effects were calculated for the combination of perceived stress and isolation (%R^2^ = 8.44%) and the combination of perceived stress, perceived social support, and isolation (%R^2^ = 20.91%). The combination of perceived stress and social support accounted for only 5.07%, and perceived social support and isolation accounted for 6.52% of the total variance in partner well-being. None of the control variables, including age, gender, duration of residence in the host country, and frequency of relocation, demonstrated statistical significance when applied in combination with the stressors.

**Table 5:** Commonality analysis representing the variance in well-being explained by perceived stress, perceived social support, loneliness, and perceived cultural distance.

Effects	Predictors	Commonality coefficient (R^2^)	*β*	*p*
Unique effects (U)	PSS	0.16	−0.46	0.001***
	FSozU-K6	0.00	0.08	0.19
	RUCLA	0.05	−0.29	0.001***
	BPCDSD	0.02	−0.14	0.006**
Common effects (C)	PSS, FSozU-K6	0.03	–	–
	PSS, RUCLA	0.05	–	–
	PSS, FSozU-K6, RUCLA	0.11	–	–

PSS, Perceived Stress Scale; FSozU-K6, Perceived Social Support Questionnaire (short version); RUCLA, Loneliness Scale (short version); BPCDSD, Brief Perceived Cultural Distance Scale. *p < 0.05, **p < 0.01, ***p < 0.001.

## Discussion

To our knowledge, this is the first study to investigate the unique and shared effects of different non-work expatriation stressors on the well-being of expatriate spouses/partners. Our findings indicate that perceived stress, perceived social support, isolation, and perceived cultural distance each contribute uniquely and jointly to the well-being of expatriate spouses and partners.

Consistent with existing research on the link between stress and well-being (Anderzén and Arnetz, 1997) and the adjustment process (Rosenbusch and Cseh, 2012), our findings revealed that perceived stress has the most significant unique impact on the well-being of expatriate partners, accounting for around 30% of the variance. This finding underscores the profound impact of stress on expatriate spouses and partners during a posting abroad, highlighting the critical need for targeted stress reduction interventions for accompanying partners.

In line with previous research on the dominant stressors of expatriation (Brown, 2008; Doki et al., 2018; Rosenbusch et al., 2015), isolation emerged as the second most significant predictor, accounting for 9.88% of the variance in partner well-being. Expatriates face isolation in several ways: leaving a well-connected environment, with proximity to family, a familiar language, and local knowledge, to relocate to a new location where these factors are absent. Additionally, the new professional role of expatriate employees may leave partners or spouses without contacts, careers, and support, thereby increasing the perception of isolation, especially among newly arrived expatriates.

In contrast to expectations, a lack of perceived social support had a low unique impact on the well-being of expatriate partners (0.0040). Nevertheless, some significance of this variable is conveyed through its interconnections with the other variables. When combined with perceived stress and isolation, the lack of perceived social support accounted for 20.91% of the total variance in partner well-being. This suggests that the social support received by expatriate partners can mitigate the adverse effects of stress and isolation, thereby improving their well-being.

When combined, perceived stress and isolation jointly contributed 8.44% to the variance in partner well-being, reinforcing the intertwined nature of the examined factors. The joint effect of perceived stress, perceived social support, and isolation (20.91%) highlights the complex interaction of these factors, suggesting a holistic approach that addresses multiple facets simultaneously for the most effective support interventions for expatriate partners and spouses.

Perceived cultural distance accounted for a marginal amount of variance in partner well-being (U = 0.02; C = 0.03), indicating that stress and isolation may play a more significant role in this population than cultural factors. This limited contribution could also be influenced by the sample composition, which predominantly comprised participants who relocated between culturally similar Western countries. Such transitions typically involve fewer cultural adjustments, leading to a diminished perception of cultural distance. Furthermore, while participants experienced considerable changes in their geographical surroundings, the effects on their immediate personal environment are likely more nuanced and intrinsic, resulting in minimal impact on their overall well-being. Nonetheless, as societies become increasingly culturally diverse, it is essential to consider the implications of cultural contexts and their potential effects on partners’ experiences and well-being.

In conclusion, our study aligns with the current literature on expatriation stress, emphasizing the roles of perceived stress, perceived social support, isolation, and perceived cultural differences. While previous investigations have consistently identified occupational stress as having a strong relationship with well-being and adjustment (Rosenbusch et al., 2015), our focus on non-work-related expatriation stressors enriches the understanding of this field and offers valuable insights into the overall adjustment process of expatriate partners.

### Implications of study findings

The findings of our study reinforce and extend the existing empirical evidence on the effects of expatriation stressors on spouses and partners, highlighting their significant impact on overall well-being (Brown, 2008; Foyle et al., 1998; Rosenbusch et al., 2015). While support interventions have been shown to effectively improve adjustment and mitigate expatriation risks (Ali et al., 2003; Cole, 2011; Lazarova et al., 2015), the lack thereof has been identified as a primary and underreported reason for assignment failure (Cole and Nesbeth, 2014). Therefore, the results of this research underscore the urgent need to complement, customize, and strengthen existing interventions to address the specific challenges faced by accompanying spouses and partners. Drawing on our findings and previous studies, we outline the following suggestions to enhance the effectiveness and sustainability of existing interventions.

#### Improving the selection process for expatriate candidates and partners

One of the most significant predictors of early termination of expatriate assignments is the difficulty that the accompanying spouse or partner experiences in adjusting to life abroad (Andreason, 2008). Therefore, the selection process should extend beyond the employee to include a comprehensive assessment of the partner’s potential stressors and adjustment capacity. Relevant factors may include the existence of a meaningful career in the home country and the feasibility of continuing or resuming it in the host country, as well as ongoing family obligations in the home country, such as caregiving responsibilities for elderly relatives. Identifying such challenges early enables organizations to make more informed decisions and tailor their preparatory support accordingly. Including the spouse or partner in the screening process ensures better alignment between the family system and the demands of international relocation, ultimately reducing the risk of failed assignments and premature returns (Andreason, 2008).

#### Designing specific interventions to target perceived stress

Our study underlines the crucial need to consolidate a discussion around stress as a risk factor and stress management as an essential topic for the entire expatriate family. Given that perceived stress accounts for the largest proportion of variance in partner well-being, it is essential to proactively increase awareness among these families about stress-related risks. The inclusion of stress management skills training should be treated as a non-negotiable component of pre-departure training, emphasizing techniques such as mindfulness-based stress reduction, cognitive-behavioral therapy, and relaxation methods. Wellness programs tailored to the specific needs and circumstances of the posting (Rosenbusch et al., 2015), and access to counseling services and support groups can offer ongoing assistance throughout the assignment, improving adjustment and decreasing stress (Platanitis, 2017). These efforts may also reduce reciprocal effects on family members through spill-over and crossover effects.

#### Addressing social isolation and promoting social support systems

With isolation emerging as the second most significant predictor of partner well-being, it is vital to support expatriate partners and spouses in fostering social connectivity to mitigate feelings of isolation (Brown, 2008). Although perceived social support contributed marginally to an individual’s well-being, its significance in combination with other variables underscores its importance. Implementing community-building programs, introducing new families to existing networks, arranging partner contacts even before relocation, or providing online support groups for expatriate partners and spouses of the same employer or organization can help alleviate feelings of isolation and establish social connections (Rosenbusch et al., 2015).

Empirical studies support the effectiveness of these interventions. Participation in online support groups has been linked to reductions in social isolation and loneliness over time, particularly when participants engage actively with the community (Trail et al., 2020). Canhilal et al. (2022) further found that virtual support platforms function as meaningful complementary resources. The availability of socioemotional support has been positively associated with better interaction and work adjustment among expatriates, highlighting the importance of emotional and informational resources in facilitating adaptation (Sterle et al., 2018b). Through sustained funding from policymakers, the impact of these support systems can be maximized. Funding for community programs, online support options, and training in networking skills can create opportunities for partners to connect and build relationships (Brown, 2008).

Employers also play a crucial role by offering flexible work schedules to employees who have recently transferred abroad with their families, thereby providing the best possible support for partners while minimizing additional stress through crossover impacts. Introducing both virtual and physical support mechanisms to accompanying partners and minimizing potential stressors before, during, and after a transition can be a powerful way to alleviate some of the burden of the expatriate employee.

#### Considering cultural contexts

While perceived cultural distance made a minor, yet unique, contribution to well-being in this sample, likely due to the predominance of relocations between culturally similar Western countries, cultural context remains a crucial consideration. As organizations expand their financial investments into new and profitable markets in Africa, Asia, and Latin America (UNCTAD, 2005), the likelihood of more parent country national expatriates relocating to culturally distant countries from their countries of origin will grow (Colakoglu and Caligiuri, 2008). Here, it needs to be kept in mind that the perceived degree of similarity between home and host countries has consistently been an important and decisive factor in an employee’s willingness to accept an international assignment, particularly when relocating with a partner or children (Wagner and Westaby, 2009).

While many companies and organizations already offer intercultural training, language courses, and host-country education/orientation programs as part of their expatriation strategy, our findings suggest that the effectiveness of these measures may depend on how well they address the specific stressors faced by accompanying partners. By deepening cultural knowledge and increasing situational familiarity, such programs have the potential to build confidence in expatriate partners, which in turn may reduce uncertainty and perceived stress. Tailored interventions that acknowledge the family’s unique composition and specific destination context can thus serve not only to ease cultural adjustment but also to strengthen psychological resilience and overall well-being during the assignment.

#### Supporting partner well-being through holistic approaches

Given the complex interplay of various predictors of the well-being of expatriate spouses and partners, a holistic and systemic approach is essential to support accompanying partners effectively. Integrating the measures outlined above into a coordinated support framework can significantly improve partner adjustment, satisfaction, and resilience throughout the expatriation. Existing programs should be complemented and designed to address both individual and family-level needs, combining practical guidance with psychological support. A well-rounded approach might include the following elements:

1. Involving spouses and partners in pre-assignment screenings to evaluate their suitability, motivation, and individual circumstances, while adapting preparatory measures accordingly.
2. Pre-assignment preparatory measures, including language and intercultural training, career transition planning, and professional orientation. These measures not only increase knowledge and familiarity with the host environment but also foster a sense of competence and reduce anticipatory stress. Additionally, training in networking strategies can strengthen social self-efficacy and help partners build meaningful connections abroad.
3. Ongoing in-country support, such as access to counseling services, peer support groups, and financial or logistical support for community engagement initiatives. These interventions help mitigate social isolation, sustain emotional well-being, and create opportunities for integration and empowerment.
4. Post-assignment debriefings, which allow partners to reflect on their experiences, consolidate gains, and foster motivation and psychological readiness for future expatriate deployments.

By addressing emotional, social, cultural, and logistical needs across all phases of the expatriation journey, such holistic programs not only enhance individual well-being but also strengthen the overall success and sustainability of international assignments.

#### Policy implications

Last, but not least, policymakers and organizational decision-makers should recognize the key role of expatriate partners in the success of the expatriation process. The interaction of multiple stressors shapes the well-being of accompanying partners, and failure to address these adequately can jeopardize both family adjustment and assignment outcomes. Continued investment in policies that promote mental well-being and social integration, particularly through the holistic, preventive measures outlined above, should be considered strategic priorities. Such investments not only enhance the overall expatriate experience for families but also contribute to greater employee satisfaction, improved retention rates, and reduced assignment failure costs for the sending organization. By acknowledging and addressing the systemic nature of expatriation challenges, policymakers can help create a more sustainable and supportive framework for internationally mobile families.

### Limitations

Demographically and contextually, this study presented several limitations: more than 90% of survey responses were completed by females. While this distribution accurately mirrors the current real-world situation in which expatriate spouses and partners are predominantly female, it limits our ability to explore the stress experiences of male expatriates. Furthermore, the sample was relatively homogenous when considering participants’ relationship status, with almost 100% in a marriage or long-term relationship. This restriction bereaves us of the opportunity to delve more deeply into the circumstances and perceptions of more contemporary family models, such as single-parent households and same-sex families. Additionally, almost 90% of participants were of Western origin, specifically from Europe or North America, while around 10% represented other world regions. Nearly half of the participants (50.7%) indicated that Europe was their current host country, which may impact their stress perception and well-being, given the comparatively high standards of living in host countries, particularly in terms of safety, healthcare, and education. Furthermore, this study did not differentiate between different types of relocation, which may significantly impact the levels, nature, and dimensions of stress. For example, investigating differences in stress factors between self-initiated expatriates and those assigned by employers, as well as dual-career couples versus single-career arrangements, and families experiencing long-term separation due to frequent business travel, would offer critical insights.

### Future directions

Future research should expand on our findings by investigating and incorporating additional stress variables, such as the stress caused by losing employment due to relocation in the case of spouses or partners. Establishing the construct of expatriation stress by further exploring the most impactful stressors and the most potent combinations, and then conceptualizing the construct, would be an interesting and helpful step in supporting accompanying spouses/partners and expatriate families. Designing a targeted scale to measure the stress levels of expatriates before, during, and after relocation could provide a simple yet powerful tool to support families in need of mitigation.

Furthermore, since our population was relatively homogeneous in terms of gender and area of origin, it would be worthwhile to diversify the population to determine whether stress perception and its impact on well-being differ across various cultural and demographic groups. In particular, the experience of male accompanying partners warrants deeper investigation. Although still a minority, their numbers are gradually increasing (Haslberger and Brewster, 2008). Male partners may be more severely affected by certain stressors, such as role reversal and career discontinuity, which can challenge their identity and increase vulnerability to stress (Cole, 2011). Social norms and persistent stereotypes in some countries can limit societal acceptance of male stay-at-home spouses, and support networks remain scarce, due to their small number or gender exclusivity. As a result, male spouses may be more isolated and under-supported (Selmer and Leung, 2003), placing them at greater risk of elevated stress and relationship strain.

## Conclusion

The present study highlights the influential role of the examined stressors – perceived stress, perceived social support, isolation, and perceived cultural distance – on the well-being of expatriated accompanying spouses and partners, providing valuable insights into their relative importance. Understanding the differing weights of stressors enables employers and sending organizations to make informed decisions regarding the design and offer of training and preparation interventions. Tailored support strategies addressing the most critical stressors may have a profound impact on the well-being and adjustment of expatriate partners and their families, and, considering its vital role in the relocation process, on the success of expatriation.

## Data Availability

The raw data supporting the conclusions of this article will be made available by the authors, without undue reservation.
